# 2024: A Year in Review

**DOI:** 10.3389/ti.2024.14243

**Published:** 2025-01-14

**Authors:** Thierry Berney, Maria Irene Bellini, Louise Benning, Oriol Bestard, Christophe Masset, Beat Moeckli, Marco Maria Pascale, Nina Pilat, Mario Sabatino, Stefan Schneeberger

**Affiliations:** ^1^ Editor-in-Chief, Transplant International; ^2^ Deputy Editor-in-Chief, Transplant International; ^3^ Editorial Fellow, Transplant International

As we embark on a new year at the helm of Transplant International (TI), the time has come to reflect on our journal’s activities and discuss some of the projects we wish to develop for 2025.

Several articles published in TI have aroused considerable attention, addressing various topics in the broad field of organ replacement. Congratulations to the authors who have submitted these papers and contributed to the maintenance of the high-quality standard of the journal (Table 1).

**TABLE 1:** Top 10 2024 highest attention articles (Altmetric scores).

Outcomes of Kidney Transplants From Toxoplasma-Positive Donors: An Organ Procurement and Transplant Network Database Analysis	Butani et al. [1]
Clinical Pig Heart Xenotransplantation—Where Do We Go From Here?	Cooper and Cozzi [2]
Liver Transplantation for Intrahepatic Cholangiocarcinoma After Chemotherapy and Radioembolization: An Intention-To-Treat Study	Maspero et al. [3]
Pre-Transplant Hyperparathyroidism and Graft or Patient Outcomes After Kidney Transplantation	Rodrigues et al. [4]
Paraneoplastic Syndrome After Kidney Transplantation: Frequency, Risk Factors, Differences to Paraneoplastic Occurrence of Glomerulonephritis in the Native Kidney, and Implications on Long-Term Kidney Graft Function	Zacrocka et al. [5]
Simultaneous Heart and Kidney Transplantation: A Systematic Review and Proportional Meta-Analysis of Its Characteristics and Long-Term Variables	Sampaio et al. [6]
Multi-Centre UK Analysis of Simultaneous Pancreas and Kidney (SPK) Transplant in Recipients With Type 2 Diabetes Mellitus	Owen et al. [7]
Public Opinions on Removing Disincentives and Introducing Incentives for Organ Donation: Proposing a European Research Agenda	Ambagtsheer et al. [8]
European Society for Organ Transplantation (ESOT) Consensus Statement on Outcome Measures in Liver Transplantation According to Value-Based Health Care	Carbone et al. [9]
Survival Advantage Comparing Older Living Donor Versus Standard Criteria Donor Kidney Transplants	Patel et al. [10]
Burnout Among Physicians of Specialties Dedicated to Liver Transplantation	Sanchez-Antolin et al. [11]

Four important special issues were completed in 2024: among them, “Living well after transplantation,” a collection focusing on various aspects that make quality of life so much improved after whole organ transplantation [12] and “Current challenges and advances on Infectious Diseases in Solid Organ Transplantation,” a timely series of articles as we exit the pandemic times that have had so much impact on our field [13].

Importantly, two of the completed special issues are the product of the tightened relationships between ESOT and TI editorial board:

1) An extensive collection on Diversity, Equity and Inclusion (DEI) in transplantation has been a significant realization of ESOT and TI commitment to the DEI values [14]. The articles published address a variety of issues that require immediate attention, from gender and race equity [15–17] to access to transplantations in underprivileged populations [18–20] or low or middle income countries [21, 22].

2) The long-awaited series of ESOT guidelines [23], written after a rigorous process during the Transplantation Learning Journey meeting held in Prague at the end of 2022 [24], are now fully available and range from oncological issues in liver transplantation [25, 26], to machine perfusion [27, 28] and biomarkers [29–31], and will undoubtedly be of regular utility and use for transplant surgeons and physicians.

All this publishing activity would not have been possible without our team of executive, associate and statistical editors, the staff at TI editorial office and the project manager from the ESOT office, Ms. Ketevan Rukhadze. Above all, the contribution of the reviewers who have spent their time and expertise to assess the papers submitted to our journal is gratefully acknowledged and we thank them for their work and voluntary participation to TI. The list of the reviewers who have contributed to TI in 2024 appears at the end of this editorial (Appendix A1).

Transplant International is proud to have started an editorial fellowship program, in which five young investigators, from different countries and professional background, have been closely associated with one of the editors-in-chief, to learn and assist in the editorial activities. In their final report, all have emphasized the educational value of the experience, the better understanding of the mechanisms of scientific publishing they got from it and the career progression it has represented for them. Editorial fellows were also engaged in communication projects for the journal and have been instrumental in creating or developing newsletters for the ESOT website, “tweetorials” and a live and interactive journal club, in which the authors and invited experts discuss selected articles recently published in TI. We have welcomed a new group of editorial fellows this past fall and we are excited to develop new projects with them. Meanwhile, we thank Chiara Becchetti, Saskia Bos, Fabian Eibensteiner, Mehdi Maanaoui and Tudor Moisoiu for their engagement and we wish them the great career in transplantation they deserve.

The biennial ESOT Congress will take place in London in 2025, from June 29 to July 2, and Transplant International will actively participate in the congress activities. Sessions designed and organized by the editorial board and editorial fellows will discuss some of the abstracts presented during the congress as well as a selection of articles recently published in TI and covering each of the five tracks defined by the scientific program committee. A “meet the editors” workshop, aiming at the younger delegates will discuss in an interactive format several aspects of scientific publication that are not always obvious for young investigators. Finally, we will publish toward the end of the year a collection of original articles based on the best communications presented at the congress.

2024 has been a busy and productive year, but more will come the way of TI readership in 2025. A collection on xenotransplantation is nearing completion and will be finalized in early 2025. We are preparing two new special issues on very hot topics in our field, namely, how transplantation practices must evolve to better serve an ageing population and the challenges and opportunities -and potential threats- of artificial intelligence when applied to our field.

From the beginning of our tenure, it has been our stated aim to knit tighter together the links between TI readership and ESOT membership. We hope that an ever-improving journal will contribute to these two communities realizing that they really are, or should be, the same and that we are stronger together in making the field of transplantation progress in Europe and beyond.

## Appendix

**TABLE A1:** Reviewers for transplant international – 2024.

Davide A. Abate
Krishna Agarwal
Kaisa Ahopelto
Emin Baris Akin
Ayman Al Jurdi
Ammar Al Midani
David Peter Al-Adra
Andreas Albertsen
Carlo Alfieri
Hakim Ali
Marc Antoine Allard
Ian Alwayn
Kristina Andrijauskaite
Madonna Rica Anggelia
Rahul Argula
Olivier Aubert
Federico Aucejo
Fahad Aziz
Daniel Azoulay
Friederike Bachmann
Rafael Badenes
Nicolas Barros Baertl
Richard James Baker
Amit Banga
David A Baran
Louise Barbier
Adam Barlow
Ivan Barone
Yuri Battaglia
Sara Battistella
Sonja Beckmann
Hanne Beeckmans
Alilis Fontana Bellorín
Alberto Benazzo
Giuditta Benincasa
David Bennett
Ilies Benotmane
Stefan Philip Berger
Michiel G.H. Betjes
Abu Bakar Hafeez Bhatti
Luigi Biancone
Christopher Blosser
Georg Böhmig
Cecilia Bonazzetti
Yuri Boteon
Rita Bottino
Dawn Bowles
Ana Cristina Breithaupt-Faloppa
Matteo Breno
Olivier Brugiere
Antoine Buemi
Leonid Bunegin
Patrizia Burra
Kadir Caliskan
Chris Callaghan
Jasper Callemeyn
Luis Camargo
Diego Cantarovich
Javier Carbone
Benno Cardini
Fatma Cebeci
Michael Cecka
Leonardo Centonze
Miriam Cortés Cerisuelo
Laurens J. Ceulemans
Ernest G Chan
Xavier Charmetant
Julien Charpentier
Xingxing Cheng
Toyofumi Chen-Yoshikawa
Aravind Cherukuri
Chi Yuen Simon Cheung
Wisit Cheungpasitporn
Umberto Cillo
Arielle Cimeno
Marc Clancy
Maarten Coemans
Luc Colas
Thomas Combriat
Laura Cosmai
Andrew Courtwright
Lionel Couzi
Peter Cowan
Emanuele Cozzi
Kristopher Croome
Elena Cuadrado-Payán
David Cucchiari
Madison Cuffy
Zoltan Czigany
John Dark
David Ross Darley
Marieke T. De Boer
Riccardo De Carlis
J. L. Campo-Cañaveral De La Cruz
Marc De Perrot
Luca Salvatore De Santo
Nicola De Stefano
Aiko P. J. De Vries
Caroline Den Hoed
Fungai Dengu
Olivier Detry
Sebastien Dharancy
Carlos Diaz
Fabienne Dobbels
Daniele Dondossola
Victoria Gomez Dos Santos
Pedro Augusto Reck Dos Santos
Jane Duffy
Michael Dunn
Magdalena Durlik
Antoine Durrbach
Philipp Dutkowski
Michael Eder
Per Ederoth
Verner Eerola
Jim Egan
Gunilla Einecke
Burcin Ekser
Kathrin Eller
Juliet Emamaullee
Marten Engelse
Eric Epailly
Michiel Elardus Erasmus
Dilmurodjon Eshmuminov
Dong Eun Lee
Patrick Evrard
Hana Fakhoury
Mario Fernández-Ruiz
Alberto Ferrarese
Sylvie Ferrari-Lacraz
Joana Ferrer-Fàbrega
Gottfried Fischer
Alan Wayne Flake
John Forsythe
Kevin Fowler
Jacopo Fumagalli
Vasiliki Galani
Zita Galvin
Ilaria Gandolfini
Wei Gao
Eduardo Miñambres Garcia
Dale Gardiner
Jeffrey J Gaynor
Laurette Geldenhuys
Anand Ghanekar
Luiza Ghila
Davide Ghinolfi
Pierre Gianello
Ian Gibson
Nicholas Gilbo
Patricia Ging
Emmanouil Giorgakis
Magali Giral
Peter Girman
Laurent Godinas
Hilary Goldberg
Masafumi Goto
Claire Goumard
Andrea Gramegna
Veronika Grau
John R Greenland
Sharlene Greenwood
Mark Greer
Paolo Antonio Grossi
Markus Guba
Benoit Guery
Luiz Felipe Guimarães
Gaurav Gupta
Ahmet Gurakar
Fadi Haidar
Fabian Halleck
Kieran Halloran
Hidetaka Hara
Takashi Harano
Hermien Hartog
Matthew Hartwig
Koji Hashimoto
Theresa Hautz
Wayne John Hawthorne
Marc Hazzan
Manfred Hecking
Ilkka Helanterä
Luuk Hilbrands
Takahisa Hiramitsu
Sandrine Hirschi
Cédric Hirzel
Martin Johannes Hoogduijn
Sarah Hosgood
Michael Hsin
Bernard Hübner
Syed Husain
Volkert Huurman
Franz Immer
Martino Introna
Georgina Irish
Fabio Ius
Hayato Iwase
Peter Jaksch
Dirk Jan Moes
Nichon Esther Jansen
Allison Jaure
Victoria Jernryd
Ik Jin Yun
Noble Johan
Joseph Kahwaji
Nassim Kamar
Hannah Kaminski
Raja Kandaswamy
Geeta Karadkhele
Jamshid Karimov
Thomas William Kay
Hiromu Kehara
Nicos Kessaris
Zeljko Kikic
Jan Klocke
Nikolaus Kneidinger
Simon Knight
Mladen Knotek
Alice Koenig
Naoru Koizumi
Katja Kotsch
Nicolas Kozakowski
Philipp Dominique Kron
Aleksandra Kukla
Vivek Kute
Florence Lacaille
Nils Lachmann
Quirino Lai
Baptiste Lamarthée
Jochen Lang
Stephen Ralph Large
Andrea Lauterio
Emilie Lebraud
Susan Lerner
Timur Lesbekov
Ping Li
Sandra Lindstedt
Cynthia Russell Lippincott
Nicolle Litjens
Antonio Loforte
Alessandro Loglio
Andrea Lombardi
Sarah Longnus
Michelle Lubetzky
Gaetano Lucisano
Bart Luijk
Jessica Lum
Georg Lurje
Grant Luxton
Colm Magee
Manuel Maglione
Alexis Maillard
Daniel G Maluf
Paolo Malvezzi
Roberto C Manfro
Tommaso Maria Manzia
Ili Margalit
Smaragdi Marinaki
Lorna Marson
La Salete Martins
Marco Masetti
Emma Massey
Arthur Matas
Marie Matignon
Shinichi Matsumoto
Katharina A. Mayer
Elena Mazza
David McGiffin
Francisco López Medrano
Sapna Mehta
Fabio Melandro
Madhav C Menon
Karsten Midtvedt
Miłosz Miedziaszczyk
Marc Giménez Milà
Irena Milaniak
Robert Minnee
Shuji Miyagawa
Sumit Mohan
Muhammad M. Mohiuddin
Irfan Moinuddin
Dina Leth Møller
Bjarne Kuno Møller
Enrique Montagud-Marrahi
Greg Moorlock
Maria Cristina Morelli
Francesc J Moreso
Ryuji Morizane
Nicolas Müller
Marcel G. Naik
Daisuke Nakajima
David Navarro
Arne Neyrinck
Christina Nguyen
Silke Niederhaus
Andriana Nikolova
Espen Nordheim
Johan Nordström
René Novysedlák
Zhuldyz Nurmykhametova
Toshihiro Okamoto
Radu Olariu
Graziano Oldani
Mihai Oltean
Francesco Orlando
Michaela Orlitova
Ivan Ortega Deballon
Fernanda Ortiz
Frank D’Ovidio
Yasemin Ozluk
Duilio Pagano
Angelica Pagliazzi
Alessandro Palleschi
Rebecca Panconesi
Christina Papachristou
Sandesh Parajuli
Judith S.L. Partridge
Renato Pascale
Damiano Patrono
Tomasz Pawinski
Maddalena Peghin
Silvia Pellegrini
Andrea Peloso
David Pereyra
Vincent Pernin
Palmina Petruzzo
Gavin Pettigrew
Benedict Phillips
Federico Piñero
Jacques Pirenne
Markus Pirklbauer
Soeren Erik Pischke
Pierluca Piselli
Manuel Alfredo Podestà
Robert Pol
Wojciech G. Polak
Stephanie Pouch
Nicola Pradegan
Francesco Procaccio
Timothy Pruett
Gervasio Soler Pujol
Chethan Puttarajappa
Junwen Qu
Marion Rabant
Maud Rabeyrin
Sam Radford
Axel Rahmel
Heinz Regele
Stefan Reuter
Samy Riad
Ilaria Righi
Federica Rigo
Paul Ritschl
Ramon Roca-Tey
Maria Delgado Roel
Alvaro Rojas
Renato Romagnoli
Gianluca Rompianesi
Joke Roodnat
Lorenzo Rosso
Lionel Rostaing
Emilio Salgado
Faouzi Saliba
Elena Sandoval
Eva Santos-Nunez
Giovanna Saracino
Kazuki Sasaki
František Saudek
David Sayah
Caitlin Sayegh
Irene Scalera
Bernhard Scheiner
Linda Scobie
Jochen Seissler
Adnan Sharif
Lisa Sharkey
Nirmal Sharma
Tara Sigdel
Fernanda Silveira
Jacques Simkins
Bojana Šimunov
Animesh Singla
Antonij Slavcev
Renaud Snanoudj
Valeria Sordi
Gionata Spagnoletti
Katharina Staufer
Robert Stratta
Delphine Szecel
Nahid Tabibzadeh
Juuso Tainio
Tomohiro Tanaka
Timucin Taner
Michiko Taniguchi
Marta Tejedor
Rachel Teo
Giuliano Testa
Dries Testelmans
Rachel Thomas
Elena Ticozzelli
Jussi Tikkanen
Francesca Tinti
Valentina Tomajer
Julian Torre-Cisneros
Davide Tosi
Luca Toti
Andreas Tzakis
Christian Unterrainer
Ilija Uzelac
Kristof Van Assche
Marian C. Clahsen-Van Groningen
Hendrik Van Leiden
Jan Van Slambrouck
Iris Van Vliet
Thomas Vanhoutte
C.A. Te Velde - Keyzer
Arzu Velioglu
Massimiliano Veroux
Erik Verschuuren
Flavio Vincenti
Julien Vionnet
Jennifer Wainright
Stephen Warrillow
Brian Wayda
Bettina Wiegmann
Aaron Wightman
Martin Wijkstrom
Karl Martin Wissing
Eckhard Wolf
Cameron Robert Wolfe
Shintaro Yagi
Dafna Yahav
Zachary Yetmar
Kenneth Yong
Peter Daechul Yoon
Lorenzo Zaffiri
Andrea Zajacova
Izabela Zakrocka
Gianluigi Zaza
Yuanyuan Zhao
Lada Zibar
Maciej Zieliński
Julien Zuber
